# Dynamic effects of bilingualism on brain structure map onto general principles of experience-based neuroplasticity

**DOI:** 10.1038/s41598-023-30326-3

**Published:** 2023-02-28

**Authors:** M. Korenar, J. Treffers-Daller, C. Pliatsikas

**Affiliations:** 1 Amsterdam Center for Language and Communication, Department of Dutch Studies, University of Amsterdam, Amsterdam, The Netherlands; 2 Alzheimer Center Amsterdam, Neurology, Amsterdam Neuroscience, Amsterdam UMC location VUmc, Vrije Universiteit Amsterdam, Amsterdam, The Netherlands; 3 School of Psychology and Clinical Language Sciences, University of Reading, Reading, UK; 4 Department of English Language and Applied Linguistics, University of Reading, Reading, UK; 5 Centro de Investigación Nebrija en Cognición, Universidad Nebrija, Madrid, Spain

**Keywords:** Cognitive neuroscience, Human behaviour

## Abstract

Bilingualism has been linked to structural adaptations of subcortical brain regions that are important for controlling multiple languages. However, research on the location and extent of these adaptations has yielded variable patterns, especially as far as the subcortical regions are concerned. Existing literature on bilingualism-induced brain restructuring has so far largely overseen evidence from other domains showing that experience-based structural neuroplasticity often triggers non-linear adaptations which follow expansion-renormalisation trajectories. Here we use generalised additive mixed models to investigate the non-linear effects of quantified bilingual experiences on the basal ganglia and the thalamus in a sample of bilinguals with a wide range of bilingual experiences. Our results revealed that volumes of the bilateral caudate nucleus and nucleus accumbens were significantly related to bilingual experiences. Importantly, these followed a non-linear pattern, with increases followed by plateauing in the most experienced bilinguals, suggesting that experience-based volumetric increases are only necessary up to a certain level of bilingual experience. Moreover, the volumes of putamen and thalamus were positively predicted by bilingual experiences. The results offer the first direct evidence that bilingualism, similarly to other cognitively demanding skills, leads to dynamic subcortical structural adaptations which can be nonlinear, in line with expansion-renormalisation models of experience-dependent neuroplasticity.

## Introduction

Mounting evidence in the past two decades has shown that the cognitively demanding experience of bilingualism affects brain structure^1^. Given that the majority of the world's population is bilingual, bilingualism-induced neuroplasticity constitutes an appealing candidate for investigating how long-lasting cognitively demanding skills and experiences affect brain morphology^2^. However, existing evidence remains fragmented and inconsistent. Therefore, the nature of such adaptations remains poorly understood^3^. Here we consider two reasons for the inconsistencies in existing results. First, the widely utilised binary comparisons between monolinguals and bilinguals have now been suggested to obscure the effects of bilingualism on the brain^4^, because bilinguals are not a monolithic group when it comes to their language experiences. Therefore, here we operationalise bilingualism as a continuum of experiences^5^ to reveal the relationship between quantified bilingual experiences and the volumes of subcortical brain regions. Second, based on recent theoretical suggestions^3^, we investigate the possibility that the effects of bilingualism on brain morphology follow non-linear trajectories. Such trajectories include volumetric increases followed by decreases, similar to those that have been reported for the acquisition and use of other types of cognitively demanding skills and experiences^6^. In the subsequent paragraphs we review the available evidence on structural brain changes in the bilingual brain through the prism of recent theoretical models, which motivated the current study.

Accumulating evidence from structural studies on bilinguals confirms that brain regions subserving switching, cognitive and articulatory control, and language selection, adapt structurally following bilingual practices^7–10^. This is particularly apparent in studies looking at subcortical regions, such as the caudate nucleus, the nucleus accumbens, the globus pallidus, the putamen, and the thalamus^7,11–17^, which are the focus of this study. These structures are key for language control, especially in experienced bilinguals, and have been shown to be particularly malleable to bilingual experiences^7,11–17^. However, the results reported across studies differ significantly, both in terms of the affected structures, but also in terms of the direction of the effects (i.e., expansions vs. contractions)^1^. Such inconsistencies have posed a challenge to the field in understanding the mechanisms of bilingualism-induced structural adaptations in subcortical structures.

The Dynamic Restructuring Model (DRM)^3^ was recently devised to reinterpret these inconsistent findings. The model builds on the expansion-renormalisation model of experience-based neuroplasticity^18^, which suggests that the acquisition of demanding skills can trigger dynamic changes in brain morphology^19–21^. These changes follow a nonlinear trajectory, with initial local expansions at the beginning of skill acquisition, possibly reflecting the creation of new local pathways that accommodate the new skills. These are followed by contractions as experience and expertise in the new skill increases, a process that signifies the identification of the most efficient of the newly created pathways, and pruning of the rest^18^. Crucially, the expansion-renormalisation trajectory of grey matter volumes has been documented for long-term skills with sustained cognitive demands^19–21^. In other words, the trajectory of structural brain adaptations is determined by the amount of experiences with the skill^22^.

Putting this into the context of bilingualism, the DRM suggests that the cognitively challenging task of juggling two languages will also cause local volumetric increases when the individual is first faced with this challenge, which will themselves start reverting to baseline with increased bilingual experience. Indeed, evidence shows increases in the caudate nucleus, a structure central to controlling between language alternatives, in less experienced bilinguals compared to monolinguals, but no effects in other subcortical structures have been reported^7,11^. By contrast, in more experienced bilinguals, the existing evidence mainly shows volumetric increases in other subcortical structures, like the putamen and the globus pallidus, which are more involved in articulatory control^7,23,24^. This effect may be related to additional demands for language production as experience increases. Similarly, reported expansions of thalamic volumes may reflect growing need for lexical selection during production as vocabulary grows with increased experiences, since the thalamus is assumed to enable a more efficient selection mechanism^25^. On the other hand, in a longitudinal study, continuous immersion in bilingual contexts have been shown to lead to reductions of volumes of the caudate nucleus^26^. This finding suggests that the initial volumetric increases of the caudate nucleus at lower levels of experience may eventually revert after extensive bilingual experience. In even higher levels of experience, such as in professional interpreters, it is possible that volumetric increases in structures such as the putamen and the thalamus also revert to baseline volumes^27^. In all, if viewed through the lens of the bilingual experiences of the samples tested, the existing evidence corroborates the suggestion by DRM: that bilingualism seen as a dynamic spectrum of experiences can bring about non-linear effects on brain structure. Nevertheless, this evidence comes from comparisons between monolinguals and groups with variable amounts of bilingual experience, so the hypotheses of non-linear effects with increasing bilingual experiences remain to be directly tested. The DRM provides a suitable and testable theoretical framework that may be able to account for divergent empirical findings, and that allows for the study of bilingualism as a dynamic experience without the need for monolingual control groups^28^.

Recent studies have provided justification for both the continuous approach to bilingualism and for the non-linearity of bilingualism-related effects on the brain. As for the former, Deluca and colleagues^12^ used the scores derived from the Language and Social Background Questionnaire (LSBQ)^5^ as continuous predictors of brain restructuring. LSBQ provides continuous measures subsuming bilingual use in different contexts, language proficiency, and switching practices, to quantify bilingual experiences. The study focused on brain *shape* changes (which may relate to volume) and revealed that social language use related to expansions of the left nucleus accumbens, caudate nucleus and right thalamus, whereas age of language acquisition predicted reshaping of the right caudate nucleus (both expansions and contractions in different parts the structure), expansions of the right putamen, and contractions in the bilateral nucleus accumbens and the thalamus^12^. These results support the notion that bilingual experiences assessed on a continuum can indeed reveal complex patterns of brain adaptations. Critically however, these analyses assumed linear structural adaptations, disallowing for testing of predictions that demanding experiences can have non-linear effects on brain structure, and, as a result, not fully testing the predictions of the DRM. Evidence for non-linear effects of bilingualism comes from a recent study looking at neurochemical indices of changes in brain morphology^29^. Using Generalised Additive Mixed Models (GAMMs), Pliatsikas and colleagues^29^ revealed non-linear effects of bilingual experiences as measured by the LSBQ on concentrations of brain metabolites in the basal ganglia. The metabolites under study were myo-Inositol and N-acetyl aspartate, markers of neuroplastic processes, including pruning^30^. The authors interpreted this as indirect evidence at the microscopic level for experience-based restructuring of the basal ganglia which depends on the amount of bilingual experiences. However, it remains to be determined whether such non-linear adaptations can also be detected at the level of volumes of relevant brain structures.

### The present study

The present study aims to shed new lights on the relationship between quantified bilingual experiences and volumes of subcortical structures involved in language acquisition and control. Previous results on subcortical neuroplasticity in bilinguals have been inconsistent. Here we employ a novel combination of methodological choices and theoretical frameworks to address the inconsistencies. First, we used GAMMs as the most appropriate method to model non-linear effects of bilingual experiences on volumes of basal ganglia and thalamus. This allows us to directly test the hypothesis that the general principles of experience-based expansion-renormalisation also apply to bilingualism-induced brain plasticity. Second, we investigated a large sample of bilinguals with a wide range of bilingual experiences, whilst keeping the languages spoken constant (see under Methods for details). We captured the dynamicity of bilingualism as a continuum by using the LSBQ composite score (henceforth Bilingual composite score; BCS). Finally, given the strong evidence that age affects the volumes of subcortical structures^31,32^, and also that bilingualism may affect each brain hemisphere differently, as far as our structures of interest are concerned^14,33^, our models controlled for age and hemisphere by adding them as predictors.

Based on the DRM, we predicted distinct trajectories of volumetric adaptations of basal ganglia and thalamus as a function of quantified bilingual experiences. Specifically, we expected an expansion-renormalisation pattern of the caudate nucleus, expressed as a trajectory suggesting increases in volume with limited bilingual experience, which will however plateau and eventually decrease as bilingual experience increases. A similar pattern was also predicted for the neighbouring nucleus accumbens, a region that is strongly interconnected with the caudate nucleus and has also been shown to contract in experienced bilinguals^12^. Furthermore, we predicted that the volumes of the putamen would be positively correlated to bilingual experiences. In contrast to the caudate nucleus and the nucleus accumbens, we did not predict the putamen to show renormalisation patterns, in line with the DRM. If the putamen manifested renormalisation, we predicted that this would occur only in individuals at the highest end of the spectrum of bilingual experiences. For the globus pallidus, we expected patterns comparable to those for the putamen due to the interrelatedness of both structures, and their shared functionality^11^. Finally, and in line with the DRM’s predictions, we expected volumes of the thalamus to correlate with bilingual experiences, with possible renormalisation patterns only in bilinguals at the highest level of bilingual experiences.

## Results

### Effects of bilingual experiences on volumetric changes in left and right hemisphere

In the first-level model using GAMMs, we examined whether bilingual composite scores had different effects on the volumes of the ROIs in the left and the right hemisphere. To do so, we examined whether BCS x Hemisphere interaction is reliably significant when its effects are tested for both the left and the right hemisphere as reference levels. For the globus pallidus, the results revealed that the BCS x Hemisphere was reliably significant with both reference levels of hemisphere (see Supplementary Materials 3). This suggested that effects of the BCS on each hemisphere of the globus pallidus may be significantly different. Therefore, in the subsequent second-level model (see next section), we split the globus pallidus data across hemispheres and examined effects of the BCS on each hemisphere. For all other ROIs, the BCS x Hemisphere interaction did not emerge as a reliably significant predictor, so we collapsed the data across hemisphere for these structures at the second level analysis.

### Effects of individual bilingual experiences on volumes of ROIs

**Figure 1:**
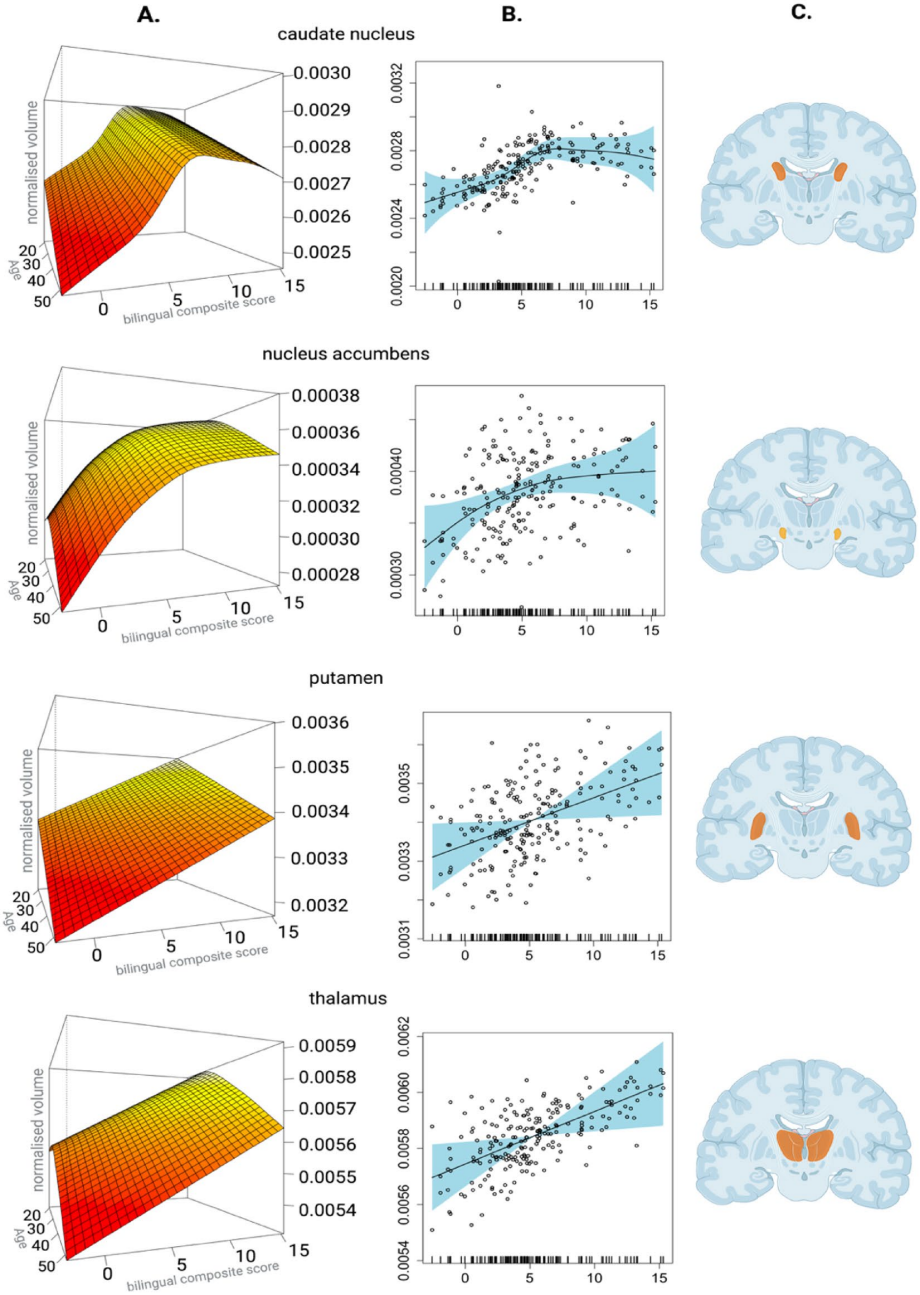
Effects of bilingual composite scores on the grey matter volumes of the ROIs. (**A**) GAMMs 3D plots illustrating the relationship between BCS (x-axes), age (z-axes) and normalised volumes of the regions of interest (rows) in cm^3^ (y-axes). (**B**) GAMMs (black line) with 95% confidence interval (blue shade) depicting the effects of the BCS scores (x-axis) and the normalised volumes of the regions of interest in cm^3^. (**C**) Featured location of the regions of interest in the brain. Created with Biorender.com.

**Figure 2:**
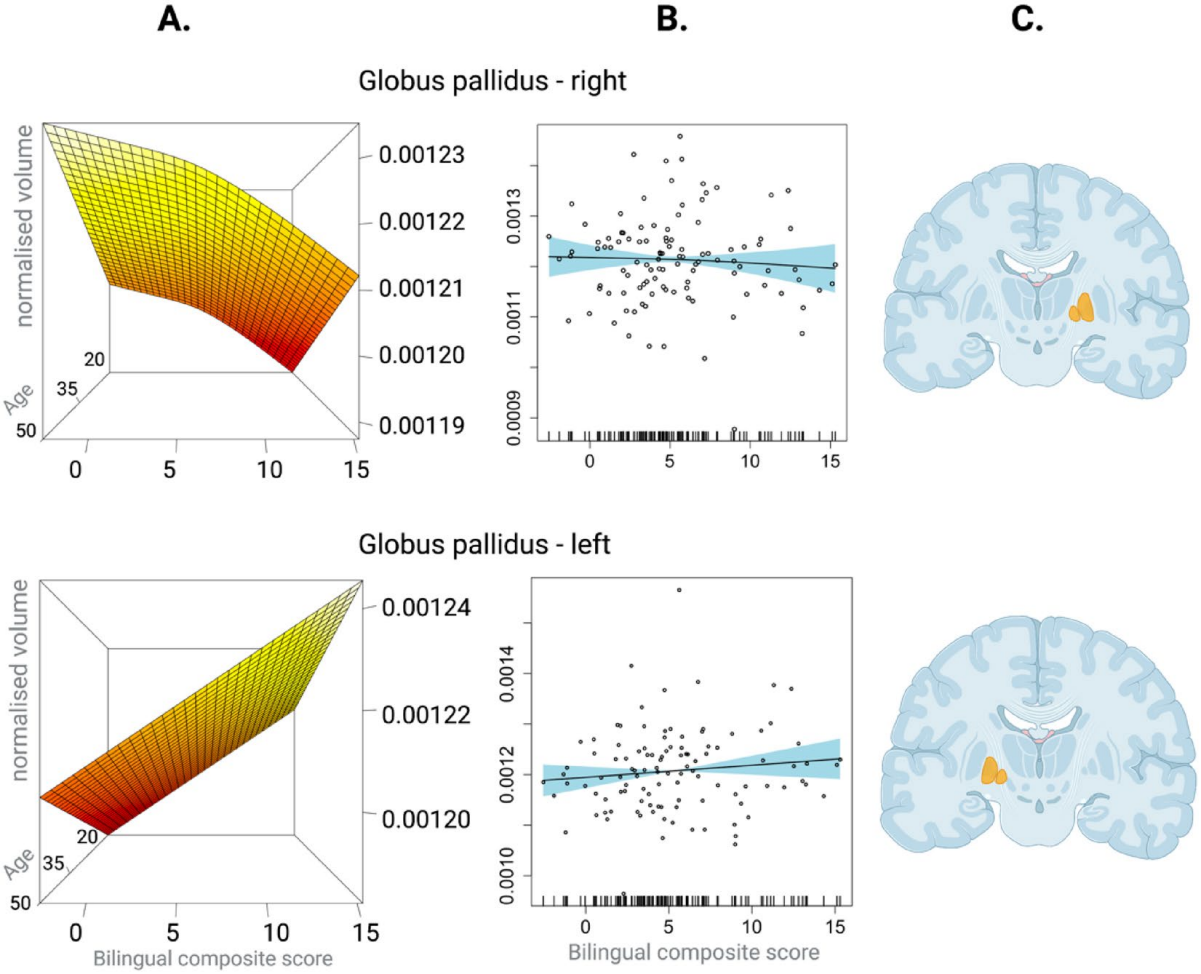
Effects of bilingual composite scores on the grey matter volumes of the globus pallidus. (**A**) GAMMs 3D plots illustrating the relationship between BCS (x-axes), age (z-axes) and normalised volumes of the globus pallidus in the right (upper band) and left hemisphere (lower band) in cm^3^ (y-axes). (**B**) GAMMs (black line) with 95% confidence interval (blue shade) depicting the effects of the BCS scores (x-axis) and the normalised volumes of the globus pallidus in cm^3^ (y-axes). (**C**) Featured location of globus pallidus in the brain. Created with Biorender.com.

The results from the second-level analysis are illustrated in Fig. 1. The BCS, the metric of interest, emerged as a non-linear predictor of volumes of the caudate nucleus and the nucleus accumbens. Specifically, results reveal a positive relationship between the BCS and the volumes in these two regions, but only to a certain level of bilingual experience after which the growth in volumes appears to reach a plateau for the nucleus accumbens and the caudate nucleus (See Fig. 1). The BCS was also a significant linear predictor of the volumes of the putamen and the thalamus, in that higher BCS levels corresponded to larger volumes. Age emerged as a significant predictor for volumes of the caudate nucleus, the putamen, and the thalamus with smaller volumes as a function of age. The analysis also revealed a significant main effect of Hemisphere. The left nucleus accumbens and the putamen were larger than the right ones. Conversely for the caudate nucleus and the thalamus, the volumes of these structures in the right hemisphere were larger than those in the left hemisphere. The two second-level models for each hemisphere of the globus pallidus showed that the BCS was not a significant predictor of volume, in either hemisphere (illustrated in Fig. 2). The results from the second second-level models for structures collapsed across hemispheres can be found in Table 1.

**Table 1:** Results from GAMMs, second-level model, with main effects of bilingual experiences (BCS), age and hemisphere, and random effects of gender and subjects on subcortical volumes of the caudate nucleus, the putamen, the nucleus accumbens, and the thalamus.

	Caudate nucleus	Nucleus accumbens	Putamen	Thalamus	Globus pallidus	
					Left	Right
BCS	< 0.001***^	0.027*^	0.028*	0.014*	0.693	0.236
Age	0.003**	0.271	0.022*	0.047*^	0.469	0.508
Hemisphere	0.009**	< 0.001***	< 0.001***	< 0.001***	–	–

Two separate models for each hemisphere for the globus pallidus with BCS and age as main effects and gender as a random effect.

For significant effects all Fs > 4.03.

Significant values are in bold.

^edf > 1.0, denoting a non-linear effect.

## Discussion

In this paper we have shown that bilingualism has dynamic effects on the volumes of the basal ganglia and the thalamus in a sample of bilinguals with varied bilingual experiences. More specifically, GAMMs analyses revealed volumetric trajectories indicating that the amount of engagement with bilingualism is linked to larger volumes of the caudate nucleus and the nucleus accumbens in less experienced bilinguals, followed by structural renormalisation in individuals with more extensive bilingual experiences. Furthermore, the amount of bilingual experiences was a positive linear predictor of the volumes of bilateral putamen and thalamus. These findings suggest that bilingualism can induce an expansion-renormalisation trajectory that is similar to what has been proposed for experience-based neuroplasticity in general^18^. The observed brain adaptations will be discussed against relevant theories and findings from previous studies below.

The positive relationship between the volumes of bilateral caudate nucleus and bilingual experiences that we observed in relatively less experienced bilinguals are consistent with previous studies. Indeed, the caudate nucleus has been reported to increase in volume mostly in bilinguals who have not been fully immersed in a bilingual environment^11^. Also, the pattern of change observed in the volumes of the caudate nucleus suggests that this structure stops growing after a certain level of bilingual experiences has been reached. Such a volumetric trajectory of the caudate nucleus can be interpreted as the beginning of optimisation of the neural resources in handling the challenges faced by bilinguals, akin to the predictions of the DRM. It is possible that with higher levels of experience the volume would even start reducing, but this does not seem to be the case for our bilinguals who reside in a non-immersive environment.

The trajectory of volumetric changes of the bilateral nucleus accumbens suggests that this structure expands as a function of growing bilingual experiences and plateaus in highly experienced bilinguals. This pattern partly matches the one for the neighbouring caudate nucleus, and it also adds to previous findings showing that the nucleus accumbens contracts as a function of length of language acquisition and expands as a function of social language use^12^. The nucleus accumbens likely subserves the reinforcement of learning strategies^34^, and larger volumes of the nucleus accumbens have been reported in individuals with more extensive social networks^35^. The plateauing pattern reported here might reflect the possibility that when bilinguals reach the necessary efficiency in language use, the intrinsic motivation to seek social interaction in their L2 stabilises. This might be particularly true for participants in the present study who stayed in their home country, where their L2 communicative skills are less important for their social wellbeing. However, given that the role of the nucleus accumbens is not well understood in bilingualism, we remain cautious of this interpretation.

With respect to the neighbouring putamen, we observed a bilateral linear volumetric increase as a function of bilingual experiences, which is consistent with previous studies and with our predictions. Larger putamen volumes have also been reported for bilinguals with long and/or intensive experiences compared to monolinguals^7^, or less experienced bilinguals^12,23^. The putamen is assumed to subserve articulatory control in selecting the appropriate motor schemata in speech production^36^. Therefore, the current results possibly indicate structural changes toward increased efficiency in L2 production.

The positive correlation between the volumes of the thalamus bilaterally and bilingual experiences might reflect the longstanding necessity for language selection among participants in the current sample. The thalamus is extensively connected to the basal ganglia and the medial prefrontal cortex subserving language and domain-general cognitive control^25,37^. Furthermore, emergent views highlight the thalamus’ role in shaping mental representations involved in learning and memory^38^. Accordingly, the present findings may reflect the need for bilinguals to constantly update mental representations, while such updates are needed more often with increased experience^39,40^.

In accordance with some more recent findings^12^, we did not observe any effects of bilingualism on the volumes of the globus pallidus, which has previously been shown to expand in experienced bilinguals^7,11^. A possible explanation relates to the fact that participants in our study were predominantly Czech natives who lived in Czechia. In such a monolingual context, bilinguals usually do not engage in code-switching^41^, the linguistic act of alternating between two languages within one sentence or paragraph^42^. Code-switching poses higher demands on cognitive control processes, which are assumed to be subserved by the globus pallidus, together with the anterior cingulate cortex^43^. However, further research is necessary to confirm whether engagement in code-switching is indeed linked to structural changes in the globus pallidus.

Some additional events emerged that related to the variables for which we controlled. Specifically, age emerged as a negative predictor of the volumes of the caudate nuclei, putamen, and the thalamus. Such findings are in line with an increasing body of evidence that shows that grey matter volume naturally decreases as we age^44^, which supports the view that the effects of age are independent of the effects induced by bilingualism. As for the effects of hemisphere, the volumetric trajectories of the caudate nucleus, the nucleus accumbens, the putamen, and the thalamus did not differ significantly for the left and the right hemispheres. Lateral differences in the trajectories of structural changes were suggested only for the globus pallidus, but volumes of this structure were not significantly predicted by bilingual experiences. However, we observed differences in the overall size of the structures in the left and right hemisphere, whereby the left nucleus accumbens and the thalamus were larger than their right homologues.. The right caudate nuclei and the putamen, by contrast, were larger than their left homologues. Similar patterns of interhemispheric differences were observed also in a large-scale study^49^. Studies on neuroanatomy show that lateral differences are not rare and can be influenced by a range of individual differences that remain beyond the scope of the current study, including sex, age, handedness, alcohol consumption, obsessive compulsivity, or psychotic experiences, to name a few^45–48^. The weight of such factors in influencing brain asymmetry of subcortical regions remains an empirical question and the sources of lateral differences are still poorly understood^49^, which points to an important direction for future studies. With respect to the present study, since all participants were bilinguals, these effects cannot be plausibly related to bilingual individual experiences. Moreover, the present findings cannot be directly compared to existing studies on bilingualism due to substantial differences in study designs. The existing evidence for effects of hemisphere comes either from studies which compared the overall size of structures in both hemispheres between groups (e.g., bilinguals vs. monolinguals or unimodal versus bimodal bilinguals)^7,24,50^, or a longitudinal study comparing the lateralised effect pre and post second language training^51^.

Findings from this study should be evaluated within the context of certain limitations. Our approach of treating bilingualism as a continuum of experiences remains relatively novel within the field of bilingualism, which restricts the comparability of our results to those from previous studies that used between-group comparisons. Furthermore, we note that this study was not designed to directly link brain adaptations to specific challenges that come with language acquisition at various stages of bilingual experiences. For example, the DRM assumes that a conflict between grammatical and syntactic aspects of two languages becomes prominent in bilinguals who have substantial experience using both languages. This is not to say that a conflict between languages at this level does not occur earlier. However, the DRM assumes that brain changes driven by controlling for two grammatical systems occur later and partly in different regions (e.g., the cerebellum^52^). Moreover, the DRM only reinterprets evidence from single cross-sectional studies which were not designed to test experience-related trajectories, and very rarely compared bilinguals with different levels of experience, meaning that patterns of experience-based non-linear adaptations may have been obscured. Therefore, a longitudinal study focusing on the specific bilingual tasks at various language acquisition stages and including whole-brain analyses would be necessary to fully confirm the DRM’s predictions. Such a study would also address another limitation of our investigation, namely that we used a measure of bilingual experiences at the time of data collection only. We also acknowledge that our understanding of the relative contribution of concrete individual bilingual experiences (i.e., code-switching frequency, age of language acquisition, multicultural identities etc.) on the brain structures is still limited, especially when using composite scores derived from self-reported questionnaires administered to populations with specific, and potentially unique, bilingual experiences. Future studies should aim to devise a composite score which subsumes sample-specific bilingual experiences and link them to structural brain changes, although we appreciate that such an endeavour requires large sample sizes to warrant enough statistical power. A promising way forward would be to combine samples from the structural studies which used LSBQ so far and to run a factor or multivariate analysis to establish which bilingual experiences or their combined effects have the most prominent effects on brain structure.

In conclusion, this study shows that bilingualism can trigger non-linear adaptations of subcortical brain volumes, expressed as initial expansions with limited bilingual experiences followed by renormalisation of some structures at higher levels of experience, indicating increased efficiency. Therefore, the data support the dynamic view of bilingualism-induced neuroplasticity proposed by the Dynamic Restructuring Model^3^, which posits that the intensity of bilingual experiences predicts dynamic patterns of structural adaptations. Thus, our findings constitute a methodological step toward a unifying explanation for previous work on bilingualism-induced neuroplasticity. On a broader level, the data also support the expansion-renormalisation model, which explains general principles of experience-dependent neuroplasticity^6^. Therefore, the current study puts bilingualism forward as a valuable candidate for studies investigating mechanisms of neural adaptations brought about by demanding, life-long experiences.

## Methods

### Participants

Data were obtained from native or native-like speakers of Czech (n = 114; 43 males; 71 females; mean age = 32). All the participants had a good command of English at B2 level on the CEFR or higher^53^, as measured by the LexTale test for advanced learners of English on a 0–100 scale (mean score = 83.25; range: 60–100; SD = 11.52)^53^. While our sample had a B2 level of English proficiency or higher, participants were sampled among bilingual groups with assumed variation in their relevant bilingual experiences. These included bilinguals with a relative short-term bilingual engagement, bilinguals who spent part of their lives abroad, or translators and interpreters. Thus, there was sufficient variability in the sample with respect to participants’ bilingual experiences, including age of L2 acquisition, level of bilingual immersion, proportionality of language use, and practices of mixing of both languages. All participants were right-handed, with normal or corrected-to-normal vision and without history of neurological or language disorders. All participants reported holding a university degree or being students enrolled in a university programme. This information was collected using the LSBQ (see Materials below). All participants were living in the Czech Republic at the time of testing and reported not to had visited any foreign speaking country two weeks prior testing. To minimize any effects caused by differences in typological proximity between the L1 and English^54^, all participants were native speakers of a Slavic language, with the following languages represented: Czech (n = 106), Russian (n = 4), Macedonian, Polish, Serbian, and Slovak (for each n = 1). The non-native Czech speakers were all court interpreters/translators for Czech-English, which means they needed to fulfil the conditions stipulated by the Czech legal Act on Experts and Interpreters no. 36/1967 Coll.^55^ including, native or native-like knowledge of Czech.

Four participants (native speakers of Czech) did not finish the whole scanning procedure, one participant did not complete the questionnaire, and their data were excluded from this study. The sample submitted for analyses consisted of 109 participants [39 males; 70 females; mean age (SD) = 32 (7.71); age range 18–53; mean LSBQ composite score (SD): 5.43 (3.96)].

Informed written consent was obtained from all the participants. The study received a favourable opinion for conduct by the Ethics Committee of Masaryk University (Ref. No. EKV-2020-013). All methods were performed in accordance with the relevant guidelines and regulations.

### Materials

#### Language and Social Background Questionnaire

To assess the participants’ level of bilingual language engagement, participants completed a Czech version of The Language and Social Background Questionnaire^5^. This questionnaire gathers information about the demographics, code-switching practices, language background, history, language use and proficiency in both languages. Answers to the questions marked on five-point or ten-point Likert scales are entered into an overall factor score calculator^5^, which creates LSBQ composite score of bilingual experiences, therefore assessing bilingualism as a continuous variable^29^. The bilingual composite score (BCS) has been calculated using the automated Factor Score Calculator as provided in Anderson and colleagues (2018). The BCS is a sum of weighted Z-scores for language use and proficiency factors. The BCS heavily reflects language use as indexed by the number of factors used to compute the BCS. Of the total of 43 bilingual factors included, four relate to L1 proficiency, two to L2 proficiency, and 38 to language use. The language use factors span information about the proportionality of using both languages in various contexts, during specific activities, and tap into switching practices. The BCS values can be also negative as the values can range between -6.58 and 32.32. The Czech version was translated from English and back-translated to determine the quality and equivalence of the Czech version with the source version. This questionnaire is attached as Supplementary Materials 5.

### MRI data acquisition

MRI data were collected at the Central European Institute of Technology (Brno, Czechia) on a 3 T Siemens MAGNETOM Prisma_fit MRI scanner, with a 32-channel Head Matrix coil.

We carried out high-resolution anatomical scans for registration and structural analysis (sagittal orientation, 256 slices, 0.7 mm slice thickness, voxel size 0.7 × 0.7 × 0.7 mm, acquisition matrix 246 × 256 mm, in-plane resolution 250 × 250, TE = 2.41 ms, TR = 2400 ms, inversion time 1140 ms, flip angle 8°). The acquisition of anatomical scans took about 10 min.

### Data analysis

#### MRI data preprocessing

We used the FSL_anat software pipeline^56^ to preprocess T1-weighted images. The image reorientation was done to the Montreal Neurological Institute (MNI)- 152 orientation. The images were corrected for field bias, automatically cropped, and nonlinearly registered to MNI space. The subcortical structures were extracted using the FIRST software pipeline, using the voxel-based morphometry^57^. The following structures were automatically segmented for both hemispheres separately and were visually inspected for quality of extractions: nucleus accumbens, caudate nuclei, globus pallidus, putamen, and thalamus. To account for the impact of head size on the volume of subcortical regions, we divided the volumes of each region by the whole-brain volume. These proportional volumes were then submitted to the statistical analysis. The mean proportional volumes of the regions of interest are illustrated in Table 2.

**Table 2:** Mean and SDs of the proportional volumes of ROIs.

	Mean proportional volume (SD)	
	Left	Right
Caudate nucleus	2.661 (0.226)	2.71 (0.281)
Nucleus accumbens	0.379 (0.071)	0.317 (0.071)
Globus pallidus	1.213 (0.09)	1.208 (0.082)
Putamen	3.408 (0.243)	3.348 (0.252)
Thalamus	5.844 (0.34)	5.712 (0.327)

All values multiplied by 1000 for the purposes of illustration.

#### Statistical data analysis

Data were analysed in R^58^ with generalised additive mixed models (GAMMs) using the gam() function of the mgcv package^59^. The non-linear regression splines in GAMMs are computed as the sum of simpler non-linear functions for each of the fitted variables. However, the non-linear splines are only included when there is enough evidence in the data for a curved function, because the wiggliness penalizes the estimated model fit. GAMMs compute the estimated degrees of freedom (edf), which indicate whether the predictor is in a non-linear (edf > 1) or a linear relationship (edf = 1) with the dependent variable. We ran a series of GAMMs for volumes of each subcortical region.

We followed a two-step procedure employed in previous studies using mixed models^60–62^. In a first-level model, we fitted the regression splines for the main effect of bilingual experiences using BCS along with the main effect of Age, and Participant and Gender as random effects. The smooth term of Age was included to account for previously observed non-linear age-related developmental changes^60^. To estimate both main effects and interactions, we applied an analytical procedure in line with the “vibration of effects” approach^62^. We included BCS x Hemisphere interaction, where Hemisphere was an ordered factor with two levels (left and right). We examined effects of this interaction to account for previously reported cases when the observed effects of BCS on brain volumes were lateralized^12^. For each ROI, we ran two GAMMs with both levels of ordered factors of Hemisphere as reference levels. The effects of the interactions Hemisphere x BCS were considered reliable only if they emerged significant in both relevant versions of the model with different reference levels.

In the second-level model, for the structure where interaction emerged as significant, we split the data for the left and right hemisphere and ran two models for each hemisphere with BCS and Age as main effects and Gender as random effect. For those regions where the BCS x Hemisphere interaction did not emerge as significant, we analysed the main effects of BCS and Hemisphere using data collapsed across hemispheres. These models further included Age as main effect and Gender and Participant as random effects.

#### The assessment of model fits

To assess the model fits of all the final models, we used the gam.check() function of the R package mgcv^63^. All the final models converged (convergence range: 7–10 iterations). The number of functions which gave rise to the regression splines exceeded in all cases the estimated degrees of freedom. For all continuous variables submitted to the analyses, there were no significant patterns in residuals as evaluated by the p-value above the 0.05 significance threshold. Also, the k-index was in all cases above 1 which supports the view that there were no missed patterns in residuals in our models^63^. The results of the model assessment analyses can be found in Supplementary Materials 1.

## Supplementary Information

Supplementary Information.

## Data Availability

The pre-processed data used in this study, including the code used to carry out the statistical analysis, are to be found in the Supplementary Materials.
