# Supplementary material for: Dynamic effects of bilingualism on brain structure map onto general principles of experience-based neuroplasticity
Source: Sci Rep. 2023 Feb 28;13:3428. doi: 10.1038/s41598-023-30326-3 (PMC9974958; doi:10.1038/s41598-023-30326-3)

**Dynamic effects of bilingualism on brain structure map onto general principles of experience-based neuroplasticity**

**Supplementary materials**

**Contents:**

1. Assessment of GAMMs fits
2. Code for the volumetric analysis
3. Results from the first level models
4. Data for the volumetric analysis
5. Czech version of LSBQ
6. Assessment of GAMMs fits using function gam.check() of the mgcv R package.

| Model diagnostics: LSBQ as predictor of caudate volumes (second-level model) | | | | |
| --- | --- | --- | --- | --- |
| Model: *gam(Volume ~ s(subject, bs= "re") + s(Age) + s(Gender , bs = "re") + Hemisphere + s(LSBQ), data = dat.caudate, method = “REML”)* | | | | |
| Smooth term | K (number of basis functions) | Estimated degrees of freedom | k-index | p-value of significant patterns in residuals |
| Age | 10 | 1.00 | 1.09 | 0.91 |
| LSBQ | 10 | 3.32 | 1.36 | 1.00 |
|  |  |  |  |  |

|  | | | | |
| --- | --- | --- | --- | --- |
| Model diagnostics: LSBQ as predictor of accumbens volumes (second-level model) | | | | |
| Model: *gam(Volume ~ s(subject, bs= "re") + s(Age) + s(Gender , bs = "re") + Hemisphere + s(LSBQ), data = dat.accumbens, method = “REML”)* | | | | |
| Smooth term | K (number of basis functions) | Estimated degrees of freedom | k-index | p-value of significant patterns in residuals |
| Age | 10 | 1.68 | 0.98 | 0.32 |
| LSBQ | 10 | 1.96 | 1.30 | 1.00 |

|  |
| --- |

|  | | | | |
| --- | --- | --- | --- | --- |
| Model diagnostics: LSBQ as predictor of putamen volumes (second-level model) | | | | |
| Model: *gam(Volume ~ s(subject, bs= "re") + s(Age) + s(Gender , bs = "re") + Hemisphere + s(LSBQ), data = dat.putamen, method = “REML”)* | | | | |
| Smooth term | K (number of basis functions) | Estimated degrees of freedom | k-index | p-value of significant patterns in residuals |
| Age | 10 | 1.00 | 0.95 | 0.19 |
| LSBQ | 10 | 1.00 | 1.33 | 1.00 |

| Model diagnostics: LSBQ as predictor of thalamus volumes (second-level model) | | | | |
| --- | --- | --- | --- | --- |
| Model: *gam(Volume ~ s(subject, bs= "re") + s(Age) + s(Gender , bs = "re") + Hemisphere + s(LSBQ), data = dat.thalamus, method = “REML”)* | | | | |
| Smooth term | K (number of basis functions) | Estimated degrees of freedom | k-index | p-value of significant patterns in residuals |
| Age | 10 | 1.95 | 1.10 | 0.93 |
| LSBQ | 10 | 1.00 | 1.42 | 1.00 |

1. Code for the volumetric analysis
2. First level model: Effects of bilingual experiences on volumetric changes in left and right hemisphere
3. GAMMs with Hemisphere as ordered factor, looking at LSBQ x Hemisphere interactions, examined for each level of Hemisphere separately. The same model was built for each region (here marked as region, the concrete regions tested were the following: {caudate, accumbens, putamen, pallidum, thalamus}). These only produced p values for the interaction, and were followed up with second level model for the interaction to be unpacked. Subject and gender treated as random effects.

-----

# Ordered factor for Hemisphere, and set contrast; Ref. = left

dat.region$Hemisphere <- relevel(dat.region$Hemisphere, ref= "left")

dat.region$OFHemisphere <- as.ordered(dat.region$Hemisphere)

contrasts(dat.region$OFHemisphere) <- 'contr.treatment'

contrasts(dat.region$OFHemisphere)

gam.LSBQL.region <- gam(Volume ~ s(subject, bs= "re") + s(Age) + s(Gender , bs = "re") +

OFHemisphere + s(LSBQ, by = OFHemisphere) + s(LSBQ),

data = dat.region, method = "REML")

-----

-----

# Ordered factor for Hemisphere, and set contrast; Ref= right

dat.region$Hemisphere <- relevel(dat.region$Hemisphere, ref= "right")

dat.region$OFHemisphere <- as.ordered(dat.region$Hemisphere)

contrasts(dat.region$OFHemisphere) <- 'contr.treatment'

contrasts(dat.region$OFHemisphere)

gam.LSBQR.region <- gam(Volume ~ s(subject, bs= "re") + s(Age) + s(Gender , bs = "re") +

OFHemisphere + s(LSBQ, by = OFHemisphere) + s(LSBQ),

data = dat.region, method = "REML")

-----

1. Second level model
2. If the LSBQ x Hemisphere interaction as examined above emerges significant, we look at each level of Hemisphere separately and produce separate smooths per group. Gender treated as random effects.

-----

# GAMM for the effects of LSBQ in the left hemisphere

gam.LSBQ.region.splitL <- gam(Volume ~ s(Age) + s(Gender , bs = "re") + s(LSBQ),

data = dat.region.left, method = "REML")

-----

-----

# GAMM for the effects of LSBQ in the right hemisphere

gam.LSBQ.region.splitR <- gam(Volume ~ s(Age) + s(Gender , bs = "re") + s(LSBQ),

data = dat.region.right, method = "REML")

-----

1. Second-level model looking at effects of bilingual experiences (LSBQ) on volumes of each region of interest collapsed across hemispheres. Applied on all regions for which the LSBQ x Hemisphere interaction (see above) did not emerge as significant predictor. Age was included as a smooth term (main effect) to account for natural decline in volumes due to aging. Hemisphere was used as a main effect (categorical) (to account for differences in mean volumes in each hemisphere), subject and gender as random effects.

-----

gam.LSBQ.region <- gam(Volume ~ s(subject, bs= "re") + s(Age) + s(Gender , bs = "re") + Hemisphere + s(LSBQ), data = dat.region, method = "REML")

-----3. Results from the first-level models

separate GAMs for each level of hemisphere as reference levels in each region of interest, looking at the effects of LSBQxHemisphere interaction on the subcortical brain volumes

| CAUDATE |  |  |
| --- | --- | --- |
| Reference level of Hemisphere | Left | Right |
| LSBQ | 0.001 | >0.001 |
| Age | 0.003 | 0.003 |
| Hemisphere | 0.008 | 0.009 |
| LSBQ x hemisphere | **0.375** | **0.780** |
|  |  |  |

| ACCUMBENS |  |  |
| --- | --- | --- |
| Reference level of Hemisphere | Left | Right |
| LSBQ | 0.038 | 0.047 |
| Age | 0.374 | 0.247 |
| Hemisphere | <0.001 | <0.001 |
| LSBQ x hemisphere | **0.378** | **0.868** |
|  |  |  |

| PUTAMEN |  |  |
| --- | --- | --- |
| Reference level of Hemisphere | Left | Right |
| LSBQ | 0.086 | 0.015 |
| Age | 0.013 | 0.013 |
| Hemisphere | <0.001 | <0.001 |
| LSBQ x hemisphere | **0.286** | **0.281** |
|  |  |  |

| PALLIDUM |  |  |
| --- | --- | --- |
| Reference level of Hemisphere | Left | Right |
| LSBQ | 0.553 | 0.259 |
| Age | 0.468 | 0.527 |
| Hemisphere | 0.465 | 0.465 |
| LSBQ x hemisphere | **0.0415*** | **0.0421*** |
|  |  |  |

| THALAMUS |  |  |
| --- | --- | --- |
| Reference level of Hemisphere | Left | Right |
| LSBQ | 0.016 | 0.019 |
| Age | 0.055 | 0.065 |
| Hemisphere | <0.001 | <0.001 |
| LSBQ x hemisphere | **0.918** | **0.941** |
|  |  |  |

| 4. Data for the volumetric analysis (the brain volumes are normalised and multiplied by 10000) | | | | | | | | | | | | | | | | | | |
| --- | --- | --- | --- | --- | --- | --- | --- | --- | --- | --- | --- | --- | --- | --- | --- | --- | --- | --- |
| Subject | Gender | Age | LSBQ | L_accumbens | L_amygdala | L_caudate | L_hippo | L_pallidum | L_putamen | L_thalamus | R_accumbens | R_amygdala | R_caudate | R_hippo | R_pallidum | R_putamen | R_thalamus | whole_brain_raw |
| 3777B | Female | 31 | 5.0984329 | 4.16 | 10.83 | 28.78 | 21.69 | 13.70 | 33.56 | 58.36 | 3.89 | 11.17 | 30.38 | 22.26 | 12.56 | 36.57 | 56.31 | 13176886250.00 |
| 3778B | Male | 40 | 1.2953917 | 3.61 | 10.57 | 26.36 | 25.31 | 12.43 | 33.04 | 53.02 | 2.99 | 13.08 | 26.12 | 28.54 | 11.15 | 31.95 | 55.52 | 16258503750.00 |
| 3800B | Female | 33 | 0.4818283 | 4.22 | 11.23 | 26.91 | 29.96 | 12.37 | 33.47 | 60.34 | 3.79 | 9.66 | 26.27 | 20.10 | 12.72 | 34.78 | 54.44 | 14906956250.00 |
| 3785B | Male | 27 | -2.5564636 | 2.88 | 9.07 | 24.15 | 23.85 | 12.55 | 32.41 | 55.33 | 1.79 | 9.11 | 26.46 | 25.81 | 11.80 | 34.32 | 57.70 | 14445907500.00 |
| 3686B | Female | 27 | 1.5228471 | 3.04 | 11.43 | 25.14 | 21.33 | 10.85 | 36.89 | 61.90 | 3.00 | 11.47 | 25.71 | 25.49 | 11.26 | 34.15 | 58.52 | 15879382500.00 |
| 3798B | Male | 31 | 6.1499241 | 3.82 | 10.23 | 24.09 | 24.78 | 11.36 | 33.21 | 59.82 | 3.51 | 10.34 | 26.84 | 25.88 | 12.38 | 33.38 | 58.26 | 16138001250.00 |
| 3799B | Female | 33 | 7.0661814 | 4.40 | 10.40 | 29.40 | 27.62 | 13.65 | 37.60 | 63.94 | 3.97 | 12.69 | 30.42 | 27.29 | 12.93 | 38.62 | 62.02 | 12253955000.00 |
| 3801B | Male | 30 | 3.2731557 | 2.44 | 11.32 | 24.45 | 26.70 | 11.23 | 32.59 | 55.58 | 1.75 | 14.40 | 20.06 | 27.50 | 11.16 | 32.17 | 57.83 | 16378883750.00 |
| 3802B | Female | 23 | 3.5445323 | 4.01 | 10.41 | 26.01 | 22.20 | 11.64 | 34.76 | 60.97 | 2.57 | 10.33 | 27.66 | 27.75 | 12.48 | 32.17 | 58.57 | 14015957500.00 |
| 3803B | Female | 27 | 4.5801734 | 4.11 | 10.43 | 27.03 | 25.46 | 11.57 | 30.04 | 57.59 | 3.50 | 9.55 | 24.19 | 27.39 | 12.34 | 29.91 | 55.36 | 14696036250.00 |
| 3804B | Female | 34 | -1.1236769 | 2.93 | 9.96 | 26.15 | 22.10 | 12.31 | 33.91 | 59.72 | 2.52 | 11.08 | 26.14 | 26.40 | 11.85 | 34.55 | 58.83 | 13942877500.00 |
| 3806B | Female | 51 | 4.3240945 | 4.47 | 8.82 | 23.69 | 27.76 | 12.11 | 36.95 | 54.81 | 3.50 | 9.66 | 24.52 | 30.11 | 12.19 | 34.25 | 53.29 | 13256411250.00 |
| 3807B | Male | 44 | 0.9240774 | 5.06 | 12.42 | 25.31 | 30.23 | 12.47 | 36.26 | 61.21 | 3.85 | 7.72 | 26.23 | 32.25 | 12.00 | 35.97 | 59.16 | 15834625000.00 |
| 3808B | Male | 33 | 4.7434871 | 3.89 | 11.39 | 29.46 | 26.30 | 12.56 | 36.97 | 66.89 | 2.89 | 10.29 | 31.42 | 25.22 | 13.66 | 37.73 | 62.32 | 12937053750.00 |
| 3809B | Female | 35 | 13.04884 | 4.83 | 11.77 | 27.46 | 26.90 | 11.77 | 35.64 | 58.39 | 4.38 | 13.73 | 24.95 | 25.27 | 11.91 | 35.63 | 55.77 | 13627571250.00 |
| 3810B | Female | 22 | 5.79194 | 4.65 | 8.88 | 27.24 | 23.62 | 12.13 | 38.09 | 60.42 | 3.65 | 8.04 | 32.39 | 25.47 | 12.70 | 36.37 | 59.13 | 12239878750.00 |
| 3811B | Male | 22 | 9.0103598 | 3.13 | 10.25 | 25.06 | 28.92 | 11.79 | 33.41 | 61.27 | 3.14 | 11.81 | 25.50 | 24.92 | 11.53 | 30.58 | 58.83 | 15677668750.00 |
| 3812B | Male | 45 | 4.2710582 | 2.91 | 12.23 | 22.86 | 26.61 | 12.24 | 32.30 | 55.60 | 2.38 | 11.57 | 23.23 | 26.01 | 12.20 | 32.92 | 52.98 | 15115602500.00 |
| 3813B | Female | 20 | 1.9104565 | 5.30 | 10.62 | 29.63 | 26.87 | 12.97 | 35.72 | 57.38 | 3.80 | 8.84 | 32.86 | 29.69 | 12.92 | 34.75 | 56.53 | 14287472500.00 |
| 3814B | Female | 26 | 10.562007 | 4.66 | 9.04 | 28.02 | 25.90 | 12.40 | 35.28 | 64.89 | 3.93 | 8.20 | 30.15 | 29.64 | 12.85 | 37.05 | 61.48 | 12830185000.00 |
| 3816B | Female | 29 | 2.4261103 | 4.09 | 12.03 | 24.16 | 18.96 | 12.53 | 32.88 | 58.54 | 2.44 | 9.20 | 26.99 | 21.59 | 12.32 | 29.52 | 57.90 | 14213298750.00 |
| 3815B | Female | 20 | 0.6057319 | 3.16 | 10.21 | 24.65 | 30.02 | 11.48 | 34.60 | 57.62 | 2.16 | 11.38 | 24.88 | 26.62 | 11.56 | 30.97 | 58.51 | 13215896250.00 |
| 3817B | Male | 20 | 8.9508882 | 3.98 | 11.44 | 27.77 | 26.60 | 10.90 | 33.98 | 56.84 | 3.90 | 12.55 | 27.38 | 24.82 | 10.92 | 32.47 | 56.49 | 15595335000.00 |
| 3820B | Male | 40 | 3.8787968 | 3.39 | 12.07 | 24.72 | 28.15 | 11.51 | 33.19 | 56.14 | 2.63 | 10.51 | 28.06 | 26.31 | 11.43 | 34.61 | 54.12 | 15967006250.00 |
| 3821B | Female | 44 | 4.6234428 | 4.04 | 9.25 | 23.15 | 32.17 | 10.51 | 25.90 | 55.02 | 3.11 | 11.99 | 23.07 | 23.95 | 10.84 | 25.42 | 53.96 | 15006010000.00 |
| 3823B | Female | 27 | 10.908805 | 3.96 | 10.44 | 25.64 | 28.37 | 11.60 | 31.85 | 56.52 | 3.35 | 9.90 | 27.38 | 26.32 | 11.76 | 33.38 | 54.31 | 15381843750.00 |
| 3824B | Female | 25 | -0.0363199 | 3.92 | 12.14 | 24.39 | 26.42 | 11.02 | 31.73 | 64.26 | 2.76 | 11.49 | 26.05 | 27.00 | 11.75 | 29.85 | 59.98 | 14325377500.00 |
| 3825B | Male | 28 | 12.802218 | 4.98 | 10.10 | 28.76 | 27.11 | 11.90 | 35.87 | 62.75 | 4.16 | 12.84 | 31.65 | 26.00 | 12.57 | 36.77 | 60.33 | 14836727500.00 |
| 3826B | Female | 22 | -0.3081331 | 2.87 | 8.32 | 28.42 | 26.36 | 12.77 | 40.06 | 56.82 | 2.31 | 12.53 | 30.42 | 25.95 | 12.60 | 38.88 | 57.55 | 12375076250.00 |
| 3827B | Female | 32 | 4.7749033 | 3.50 | 11.01 | 26.15 | 27.62 | 12.51 | 33.69 | 58.73 | 3.44 | 11.36 | 27.08 | 25.41 | 12.88 | 32.56 | 59.31 | 16184945000.00 |
| 3922B | Male | 31 | 5.0952573 | 3.50 | 11.01 | 26.15 | 27.62 | 12.51 | 33.69 | 58.73 | 3.44 | 11.36 | 27.08 | 25.41 | 12.88 | 32.56 | 59.31 | 16184945000.00 |
| 3872B | Female | 26 | 5.6268425 | 4.01 | 10.52 | 30.90 | 25.47 | 14.54 | 39.60 | 58.55 | 3.41 | 8.61 | 29.70 | 24.33 | 15.63 | 36.13 | 56.88 | 14604613750.00 |
| 3873B | Male | 31 | 2.3550683 | 3.68 | 9.75 | 26.90 | 29.38 | 11.81 | 32.24 | 59.22 | 3.03 | 11.37 | 26.80 | 33.95 | 11.65 | 31.44 | 58.50 | 15789433750.00 |
| 3870B | Female | 27 | 3.1983615 | 3.79 | 22.09 | 30.83 | 22.11 | 12.74 | 37.85 | 63.00 | 3.02 | 5.11 | 19.76 | 25.57 | 12.45 | 35.43 | 60.24 | 13542672500.00 |
| 3871B | Female | 27 | 6.4404076 | 3.03 | 10.86 | 28.38 | 25.28 | 11.89 | 37.26 | 59.88 | 3.70 | 10.23 | 28.64 | 29.62 | 12.06 | 36.83 | 59.82 | 13584631250.00 |
| 3828B | Male | 27 | 3.9945674 | 4.03 | 10.23 | 29.45 | 26.04 | 12.77 | 36.21 | 60.44 | 3.10 | 11.16 | 30.38 | 27.38 | 12.34 | 36.34 | 60.77 | 15080648750.00 |
| 3829B | Male | 23 | 2.0718559 | 3.67 | 9.84 | 25.32 | 25.35 | 12.59 | 35.23 | 57.62 | 3.91 | 12.18 | 23.43 | 27.15 | 11.88 | 32.80 | 54.00 | 14610938750.00 |
| 3831B | Male | 30 | 2.1060107 | 4.41 | 11.29 | 26.10 | 24.81 | 11.45 | 32.89 | 59.52 | 4.71 | 12.12 | 25.50 | 22.96 | 11.63 | 33.98 | 57.85 | 16935296250.00 |
| 3833B | Female | 21 | 11.315129 | 3.67 | 10.88 | 29.10 | 29.13 | 13.34 | 35.35 | 60.67 | 3.51 | 10.42 | 28.72 | 28.54 | 13.72 | 33.53 | 59.22 | 12604265000.00 |
| 3834B | Female | 27 | 6.7714438 | 3.64 | 11.03 | 30.34 | 24.79 | 12.06 | 34.59 | 59.26 | 2.27 | 10.13 | 32.31 | 21.70 | 11.95 | 32.80 | 58.99 | 15564496250.00 |
| 3835B | Female | 22 | 1.1599952 | 4.32 | 10.38 | 24.23 | 30.95 | 12.49 | 34.21 | 57.99 | 3.31 | 9.60 | 23.93 | 33.44 | 11.20 | 32.21 | 57.07 | 13458380000.00 |
| 3837B | Female | 25 | 2.4280809 | 3.77 | 10.86 | 26.49 | 27.42 | 10.58 | 34.05 | 63.92 | 3.82 | 11.28 | 24.84 | 22.47 | 11.77 | 33.54 | 61.50 | 13737223750.00 |
| 3836B | Female | 21 | 3.1161046 | 3.83 | 8.64 | 27.53 | 23.23 | 11.51 | 33.64 | 51.10 | 2.30 | 8.31 | 25.76 | 27.89 | 11.21 | 32.29 | 50.32 | 12505853750.00 |
| 3840B | Female | 19 | 8.9910398 | 4.57 | 7.87 | 22.61 | 20.04 | 12.02 | 37.08 | 59.25 | 3.87 | 10.21 | 27.58 | 22.59 | 10.56 | 33.93 | 57.13 | 14866405000.00 |
| 3839B | Female | 21 | 1.1796171 | 2.32 | 6.23 | 26.60 | 24.72 | 11.40 | 34.56 | 53.76 | 2.04 | 8.85 | 27.87 | 24.93 | 11.46 | 32.96 | 51.14 | 14583806250.00 |
| 3841B | Male | 33 | 0.63675 | 3.48 | 10.66 | 23.24 | 24.43 | 11.62 | 31.85 | 58.48 | 3.84 | 10.07 | 25.60 | 24.94 | 11.19 | 32.13 | 57.03 | 15079120000.00 |
| 3842B | Male | 23 | 7.1519861 | 3.34 | 9.28 | 25.79 | 21.63 | 10.10 | 30.47 | 53.41 | 2.57 | 10.07 | 25.48 | 24.82 | 10.84 | 29.83 | 53.82 | 14932745000.00 |
| 3843B | Male | 33 | 2.2769211 | 3.58 | 8.34 | 23.66 | 21.54 | 11.12 | 32.06 | 50.10 | 3.10 | 9.43 | 22.43 | 24.06 | 9.64 | 29.16 | 50.80 | 17551010000.00 |
| 3845B | Male | 35 | -1.1325613 | 3.63 | 10.36 | 25.11 | 37.23 | 13.25 | 34.46 | 61.28 | 2.19 | 13.66 | 27.16 | 25.73 | 12.14 | 32.62 | 63.95 | 14832903750.00 |
| 3846B | Female | 46 | 5.7294717 | 3.66 | 8.58 | 28.95 | 29.33 | 14.24 | 35.81 | 61.17 | 3.28 | 7.35 | 30.22 | 30.97 | 12.13 | 37.45 | 60.35 | 12115936250.00 |
| 3847B | Male | 31 | 4.3944217 | 4.14 | 12.68 | 24.21 | 27.36 | 12.24 | 31.27 | 59.85 | 2.88 | 12.09 | 25.08 | 27.66 | 11.79 | 30.79 | 61.05 | 15310558750.00 |
| 3848B | Male | 32 | 2.7385143 | 4.55 | 10.85 | 25.78 | 25.69 | 14.21 | 33.60 | 58.08 | 3.05 | 11.38 | 24.88 | 25.26 | 14.14 | 32.88 | 56.30 | 14396296250.00 |
| 3849B | Male | 33 | 3.4695821 | 2.93 | 10.70 | 25.10 | 26.82 | 11.21 | 34.26 | 53.67 | 3.24 | 10.50 | 23.82 | 25.85 | 11.71 | 33.93 | 51.60 | 17413248750.00 |
| 3850B | Male | 26 | 4.7312617 | 4.21 | 9.50 | 27.59 | 25.20 | 11.49 | 35.52 | 54.71 | 3.53 | 10.36 | 28.75 | 27.36 | 11.07 | 30.99 | 52.61 | 15407275000.00 |
| 3851B | Male | 36 | 4.3690749 | 3.37 | 9.83 | 24.63 | 24.18 | 12.30 | 30.99 | 52.47 | 2.72 | 10.74 | 22.36 | 24.58 | 11.38 | 29.15 | 50.87 | 15662422500.00 |
| 3852B | Female | 31 | 3.0925725 | 3.64 | 11.65 | 25.66 | 27.76 | 12.49 | 33.10 | 59.94 | 3.22 | 10.10 | 25.47 | 23.39 | 11.98 | 32.55 | 55.94 | 13386095000.00 |
| 3853B | Male | 40 | -1.3324693 | 2.35 | 10.85 | 21.34 | 25.02 | 10.98 | 28.31 | 52.66 | 1.19 | 17.11 | 21.93 | 24.15 | 12.04 | 29.00 | 50.61 | 17351530000.00 |
| 3854B | Female | 44 | 6.6062257 | 3.25 | 14.03 | 27.01 | 24.77 | 12.14 | 32.89 | 59.65 | 2.68 | 11.64 | 26.72 | 28.56 | 12.36 | 31.66 | 57.75 | 13675930000.00 |
| 3855B | Female | 36 | 15.115394 | 4.56 | 11.46 | 26.75 | 29.25 | 11.70 | 34.42 | 61.56 | 3.09 | 10.92 | 28.40 | 27.49 | 12.24 | 35.05 | 59.35 | 12752011250.00 |
| 3857B | Male | 31 | 12.376858 | 3.89 | 11.06 | 29.43 | 26.55 | 13.49 | 37.04 | 61.58 | 2.98 | 11.77 | 30.36 | 27.80 | 13.68 | 36.65 | 59.57 | 14118657500.00 |
| 3859B | Female | 35 | 2.0805384 | 3.42 | 9.74 | 29.31 | 31.18 | 11.95 | 35.15 | 63.86 | 3.10 | 10.24 | 32.80 | 29.46 | 13.00 | 38.38 | 59.45 | 13679446250.00 |
| 3858B | Female | 34 | 7.2552043 | 4.40 | 13.19 | 28.65 | 28.94 | 13.48 | 33.70 | 59.50 | 3.49 | 12.23 | 27.03 | 31.19 | 12.44 | 35.18 | 59.76 | 13908770000.00 |
| 3860B | Female | 31 | -1.215375 | 3.24 | 10.84 | 25.45 | 21.90 | 12.21 | 31.47 | 53.75 | 2.60 | 12.19 | 25.47 | 22.90 | 10.87 | 31.57 | 50.17 | 13373573750.00 |
| 3861B | Male | 24 | 3.3600197 | 2.94 | 9.76 | 27.30 | 26.09 | 11.58 | 34.39 | 56.09 | 3.30 | 11.66 | 26.88 | 23.71 | 11.41 | 31.21 | 55.09 | 15362238750.00 |
| 3862B | Female | 27 | 5.7296851 | 4.08 | 10.99 | 27.64 | 27.12 | 12.31 | 33.32 | 59.12 | 2.68 | 16.15 | 29.66 | 25.35 | 12.37 | 32.09 | 57.51 | 15371506250.00 |
| 3863B | Female | 25 | 5.595505 | 2.84 | 10.32 | 27.18 | 27.23 | 12.16 | 32.96 | 59.26 | 3.50 | 10.74 | 29.61 | 25.67 | 11.47 | 34.29 | 58.13 | 14483695000.00 |
| 3866B | Male | 42 | 15.308235 | 4.06 | 10.83 | 25.80 | 26.84 | 12.10 | 37.95 | 60.72 | 2.90 | 12.33 | 24.90 | 27.61 | 12.34 | 36.93 | 59.93 | 15069595000.00 |
| 3874B | Female | 28 | 7.3739955 | 3.57 | 9.88 | 31.12 | 26.22 | 12.24 | 36.80 | 58.37 | 2.73 | 13.56 | 31.14 | 27.20 | 12.55 | 35.98 | 55.42 | 13406511250.00 |
| 3875B | Female | 32 | 3.7089151 | 3.13 | 11.87 | 23.71 | 25.08 | 10.43 | 31.80 | 55.99 | 3.78 | 13.14 | 23.05 | 20.99 | 12.41 | 33.42 | 55.48 | 14244477500.00 |
| 3876B | Male | 30 | 1.8002433 | 4.46 | 10.66 | 24.99 | 23.29 | 12.47 | 31.31 | 58.14 | 3.85 | 11.52 | 25.05 | 24.76 | 12.28 | 33.55 | 55.43 | 18351481250.00 |
| 3877B | Female | 33 | 14.32236 | 3.71 | 9.41 | 27.27 | 29.96 | 11.55 | 34.86 | 59.51 | 2.84 | 10.27 | 26.84 | 30.53 | 11.60 | 35.65 | 58.47 | 12939828750.00 |
| 3878B | Male | 27 | 7.9136135 | 5.42 | 11.36 | 28.90 | 25.39 | 13.52 | 35.15 | 60.09 | 4.22 | 9.95 | 29.89 | 30.06 | 12.48 | 34.51 | 59.84 | 16116506250.00 |
| 3880B | Female | 45 | 3.8014277 | 2.95 | 11.10 | 24.49 | 25.64 | 12.04 | 31.26 | 56.54 | 3.13 | 9.07 | 25.80 | 24.12 | 11.71 | 32.43 | 52.70 | 14261973750.00 |
| 3884B | Female | 36 | 4.8462133 | 4.92 | 12.03 | 30.34 | 27.89 | 12.77 | 32.52 | 59.28 | 3.19 | 12.99 | 29.81 | 30.59 | 12.51 | 31.73 | 59.45 | 13119180000.00 |
| 3891B | Male | 25 | 6.7610098 | 4.59 | 10.38 | 32.48 | 27.51 | 12.81 | 37.23 | 60.10 | 4.44 | 11.87 | 33.20 | 28.20 | 13.77 | 36.04 | 60.35 | 13540926250.00 |
| 3892B | Male | 35 | 5.2328812 | 2.96 | 11.10 | 21.24 | 24.34 | 11.51 | 30.05 | 50.09 | 2.76 | 10.64 | 22.86 | 25.62 | 11.05 | 29.22 | 50.43 | 18077958750.00 |
| 3894B | Female | 20 | 4.7475064 | 3.24 | 9.59 | 29.43 | 29.79 | 14.01 | 38.71 | 61.58 | 2.39 | 9.88 | 30.13 | 27.29 | 12.68 | 35.64 | 60.58 | 12768852500.00 |
| 3895B | Female | 50 | 7.9543499 | 2.70 | 8.68 | 25.57 | 28.93 | 12.27 | 29.71 | 60.07 | 1.58 | 9.07 | 26.48 | 27.74 | 11.92 | 30.20 | 59.33 | 13917252500.00 |
| 3896B | Female | 35 | 13.243137 | 3.69 | 10.65 | 26.01 | 26.71 | 10.70 | 33.25 | 56.64 | 2.00 | 8.06 | 24.87 | 24.43 | 11.84 | 31.38 | 52.34 | 16415495000.00 |
| 3899B | Female | 27 | 4.9508122 | 2.39 | 8.65 | 23.82 | 28.54 | 12.37 | 32.96 | 57.88 | 3.81 | 9.25 | 24.06 | 28.50 | 12.01 | 32.70 | 54.99 | 13839688750.00 |
| 3900B | Male | 24 | 7.0045493 | 4.81 | 11.74 | 28.82 | 25.71 | 13.26 | 38.95 | 66.36 | 3.86 | 14.10 | 30.15 | 27.16 | 12.79 | 39.27 | 65.94 | 15726615000.00 |
| 3902B | Female | 24 | 4.5511747 | 3.05 | 10.75 | 27.16 | 24.88 | 11.89 | 33.08 | 61.96 | 2.82 | 11.01 | 28.12 | 26.07 | 11.33 | 33.11 | 59.24 | 12885566250.00 |
| 3901B | Female | 36 | 2.7792006 | 4.74 | 10.07 | 26.93 | 24.68 | 11.14 | 35.85 | 56.90 | 3.27 | 11.11 | 28.36 | 22.14 | 12.15 | 34.66 | 52.91 | 14486713750.00 |
| 3914B | Male | 34 | 9.0152705 | 2.80 | 9.48 | 25.66 | 28.11 | 8.79 | 31.35 | 55.52 | 1.91 | 11.30 | 25.76 | 29.19 | 10.78 | 32.08 | 54.11 | 15110647500.00 |
| 3915B | Female | 38 | 12.51729 | 4.08 | 9.95 | 27.42 | 25.39 | 12.81 | 33.88 | 58.15 | 2.92 | 10.13 | 26.72 | 25.89 | 12.22 | 31.60 | 58.08 | 15168686250.00 |
| 3916B | Female | 51 | 4.0511803 | 3.93 | 9.17 | 25.83 | 24.99 | 11.92 | 34.15 | 57.75 | 2.42 | 10.24 | 25.34 | 23.16 | 11.40 | 32.25 | 55.56 | 13311912500.00 |
| 3917B | Female | 25 | 2.9404485 | 4.61 | 8.48 | 25.93 | 22.90 | 12.04 | 35.87 | 59.81 | 3.16 | 11.51 | 28.18 | 23.41 | 12.05 | 34.91 | 57.70 | 13888066250.00 |
| 3918B | Female | 50 | 5.4658845 | 3.63 | 12.29 | 25.43 | 25.06 | 12.19 | 30.95 | 51.99 | 2.59 | 12.43 | 25.97 | 24.88 | 11.49 | 28.35 | 52.11 | 14165052500.00 |
| 3923B | Male | 44 | 10.660846 | 3.46 | 12.48 | 27.10 | 30.76 | 12.63 | 34.47 | 60.57 | 3.38 | 13.15 | 26.10 | 24.29 | 12.34 | 33.57 | 60.23 | 15794281250.00 |
| 3924B | Female | 21 | 6.104092 | 3.85 | 10.96 | 27.89 | 28.29 | 11.62 | 36.86 | 57.56 | 3.57 | 9.42 | 29.58 | 28.41 | 12.11 | 33.81 | 58.98 | 14189542500.00 |
| 3925B | Female | 20 | 9.7815679 | 3.47 | 8.31 | 27.59 | 22.00 | 11.37 | 33.96 | 54.41 | 2.00 | 7.51 | 28.35 | 22.92 | 11.10 | 32.25 | 53.45 | 13529991250.00 |
| 3926B | Female | 47 | -1.912905 | 3.26 | 10.39 | 22.88 | 27.10 | 12.27 | 34.61 | 50.60 | 3.29 | 12.52 | 22.63 | 27.02 | 11.69 | 32.56 | 48.69 | 14571451250.00 |
| 3928B | Female | 28 | 3.5419857 | 3.25 | 12.02 | 23.56 | 26.87 | 12.01 | 31.84 | 59.29 | 2.91 | 12.07 | 25.07 | 23.58 | 12.08 | 31.10 | 57.98 | 13354018750.00 |
| 3936B | Male | 45 | 13.310601 | 3.44 | 11.67 | 27.27 | 29.10 | 11.27 | 32.92 | 58.53 | 3.49 | 11.07 | 28.25 | 30.28 | 12.28 | 31.87 | 57.67 | 15792062500.00 |
| 3942B | Female | 37 | 11.124654 | 4.13 | 10.24 | 27.85 | 29.39 | 11.96 | 37.43 | 57.09 | 4.13 | 8.54 | 28.77 | 28.34 | 13.07 | 39.12 | 55.20 | 12858263750.00 |
| 3946B | Female | 50 | 3.5175616 | 2.38 | 11.34 | 23.70 | 25.31 | 12.65 | 29.34 | 53.18 | 2.20 | 12.16 | 23.35 | 29.17 | 13.09 | 31.66 | 54.97 | 12990341250.00 |
| 3947B | Female | 31 | 9.6221489 | 4.72 | 9.57 | 28.24 | 26.44 | 12.39 | 32.68 | 56.55 | 3.41 | 10.90 | 31.24 | 27.47 | 11.78 | 36.14 | 57.38 | 13567090000.00 |
| 3951B | Female | 30 | 5.5182585 | 4.74 | 10.46 | 30.78 | 26.65 | 13.02 | 32.28 | 60.42 | 4.97 | 11.97 | 30.62 | 25.97 | 11.85 | 34.33 | 60.02 | 13720502500.00 |
| 4419A | Female | 52 | 3.3741142 | 3.86 | 14.73 | 26.52 | 26.34 | 13.51 | 35.44 | 56.66 | 4.26 | 11.51 | 28.88 | 26.74 | 13.48 | 33.73 | 56.41 | 14386523750.00 |
| 3953B | Female | 23 | 6.4132301 | 5.20 | 10.11 | 27.05 | 26.66 | 11.26 | 32.48 | 58.79 | 3.69 | 8.27 | 28.43 | 30.81 | 10.87 | 31.97 | 57.28 | 15115963750.00 |
| 3956B | Female | 30 | 4.5022556 | 4.00 | 8.61 | 28.27 | 24.50 | 12.25 | 36.46 | 59.55 | 3.22 | 9.34 | 25.64 | 24.60 | 12.29 | 34.43 | 59.78 | 12122621250.00 |
| 3960B | Female | 43 | 6.0493674 | 4.47 | 7.83 | 28.97 | 28.95 | 11.66 | 34.65 | 63.56 | 3.52 | 8.08 | 30.58 | 30.69 | 12.50 | 34.37 | 60.35 | 13500315000.00 |
| 3962B | Male | 25 | 8.8020196 | 3.62 | 11.83 | 27.95 | 29.04 | 12.28 | 34.67 | 60.82 | 4.05 | 9.53 | 29.03 | 26.27 | 12.75 | 35.04 | 60.87 | 12484698750.00 |
| 3963B | Female | 36 | 5.7609734 | 4.31 | 10.88 | 28.48 | 28.80 | 13.26 | 36.53 | 61.43 | 3.42 | 11.39 | 27.10 | 26.53 | 12.57 | 36.18 | 59.62 | 13710371250.00 |
| 3965B | Male | 24 | 9.3513054 | 2.64 | 12.21 | 25.19 | 23.42 | 11.93 | 32.83 | 54.81 | 2.73 | 10.64 | 25.51 | 31.18 | 11.36 | 34.69 | 56.61 | 14606523750.00 |
| 3970B | Female | 32 | 7.0348395 | 5.04 | 8.65 | 30.54 | 29.65 | 12.25 | 36.34 | 64.24 | 4.58 | 9.70 | 30.47 | 29.77 | 11.50 | 35.09 | 61.90 | 13565320000.00 |
| 3166B | Female | 46 | 1.9809168 | 3.57 | 11.57 | 23.93 | 20.75 | 12.78 | 32.22 | 54.82 | 1.91 | 11.47 | 24.57 | 25.53 | 12.69 | 30.36 | 54.46 | 17036918750.00 |
| 3979B | Female | 37 | 0.5198432 | 4.59 | 14.37 | 25.50 | 28.96 | 12.53 | 34.90 | 60.20 | 3.91 | 11.14 | 26.64 | 28.26 | 12.47 | 33.96 | 57.06 | 13227640000.00 |
| 3982B | Female | 38 | 11.945215 | 3.62 | 11.48 | 27.51 | 27.97 | 11.52 | 32.68 | 59.63 | 3.17 | 12.07 | 29.57 | 27.61 | 11.80 | 31.92 | 57.51 | 13070257500.00 |

**5. Czech version of the Language Social Background Questionnaire**


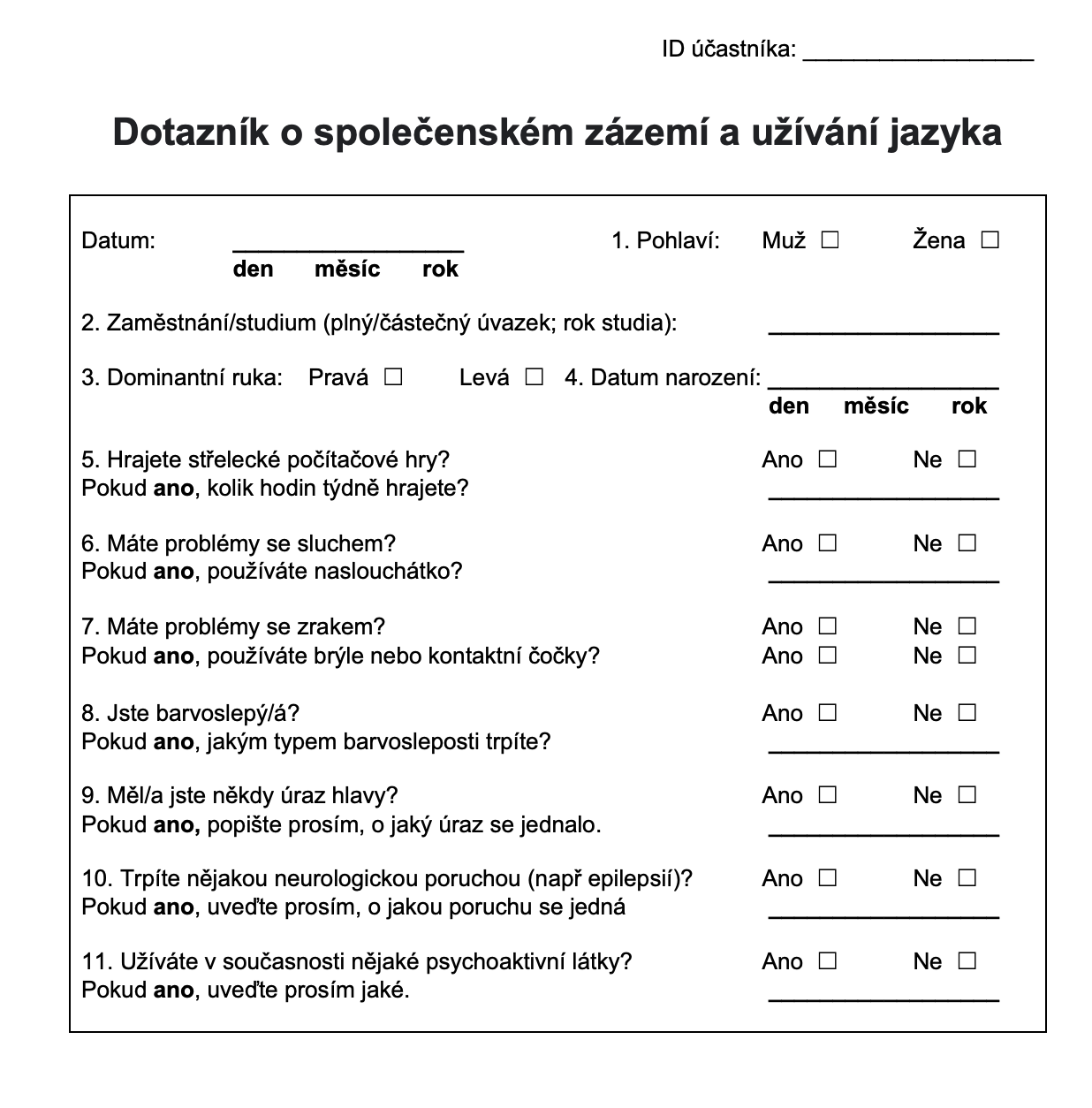


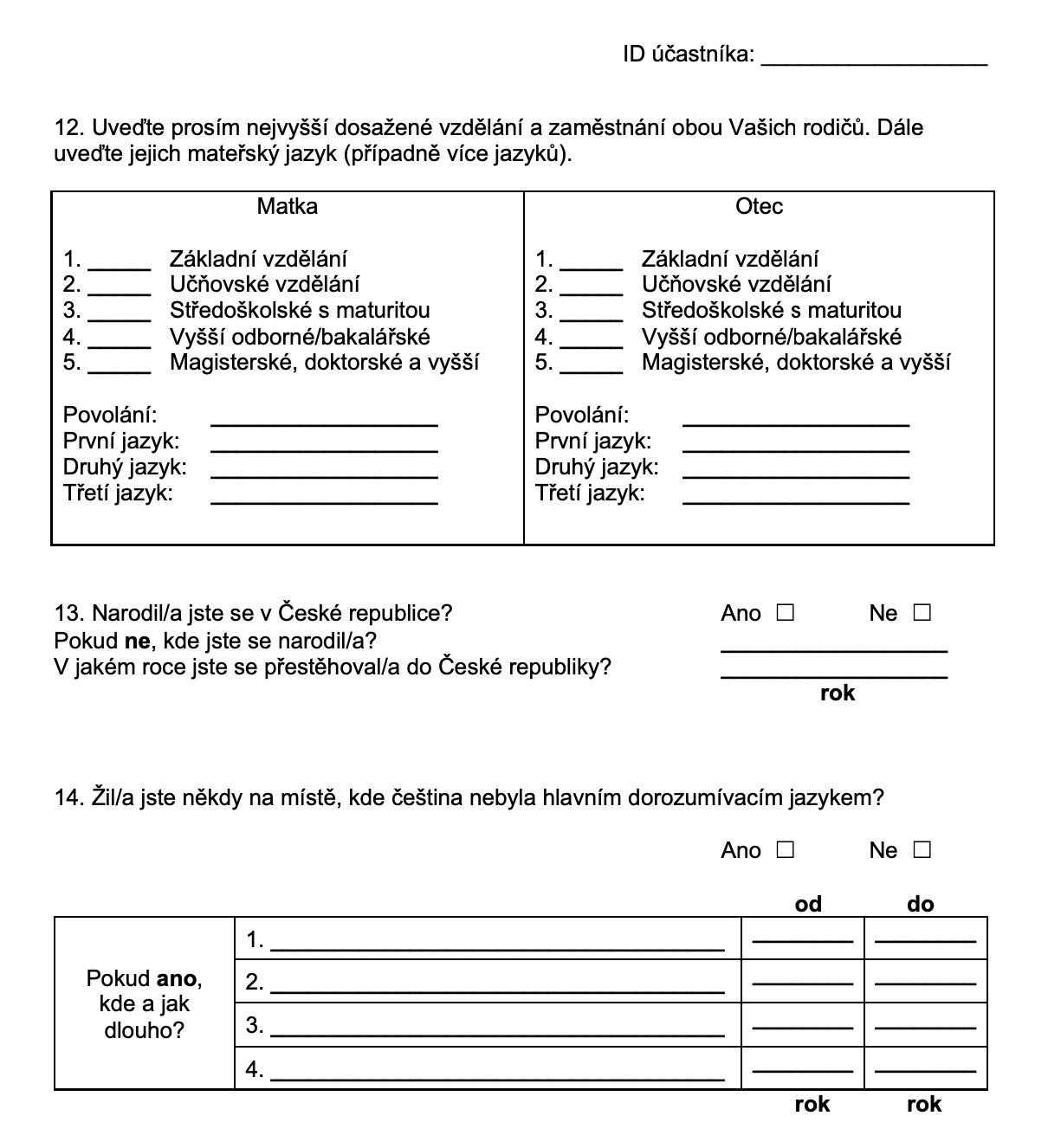


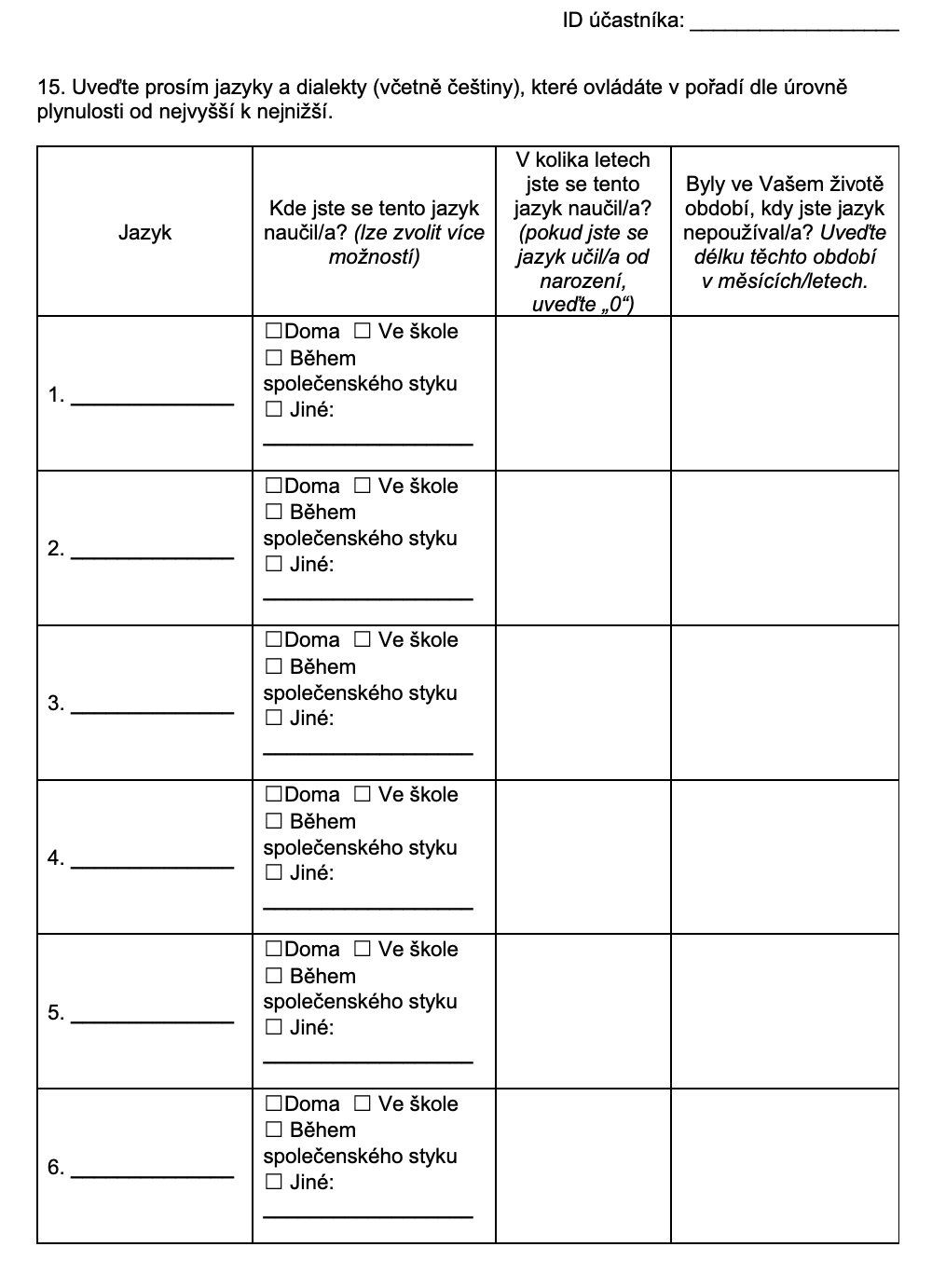


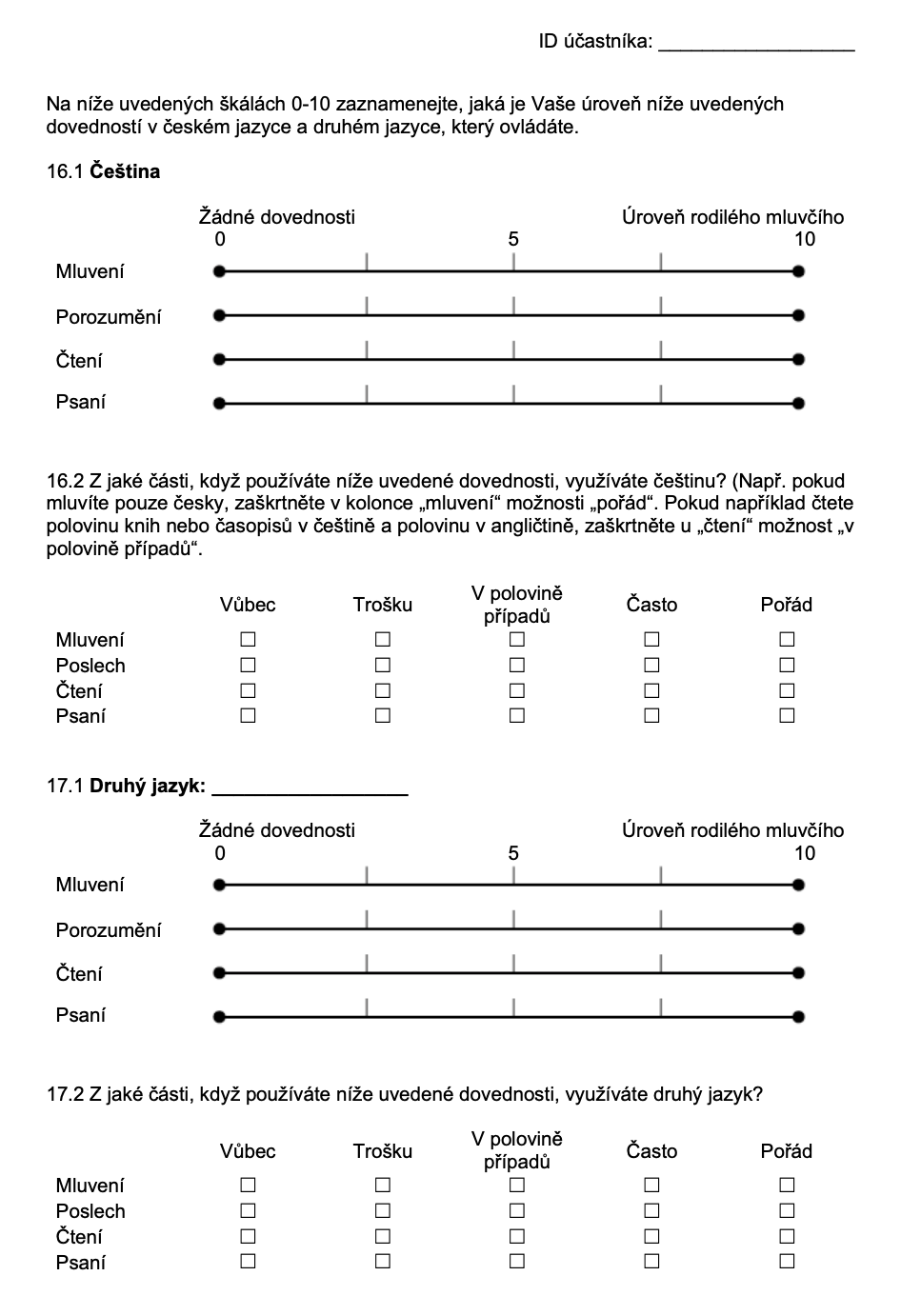


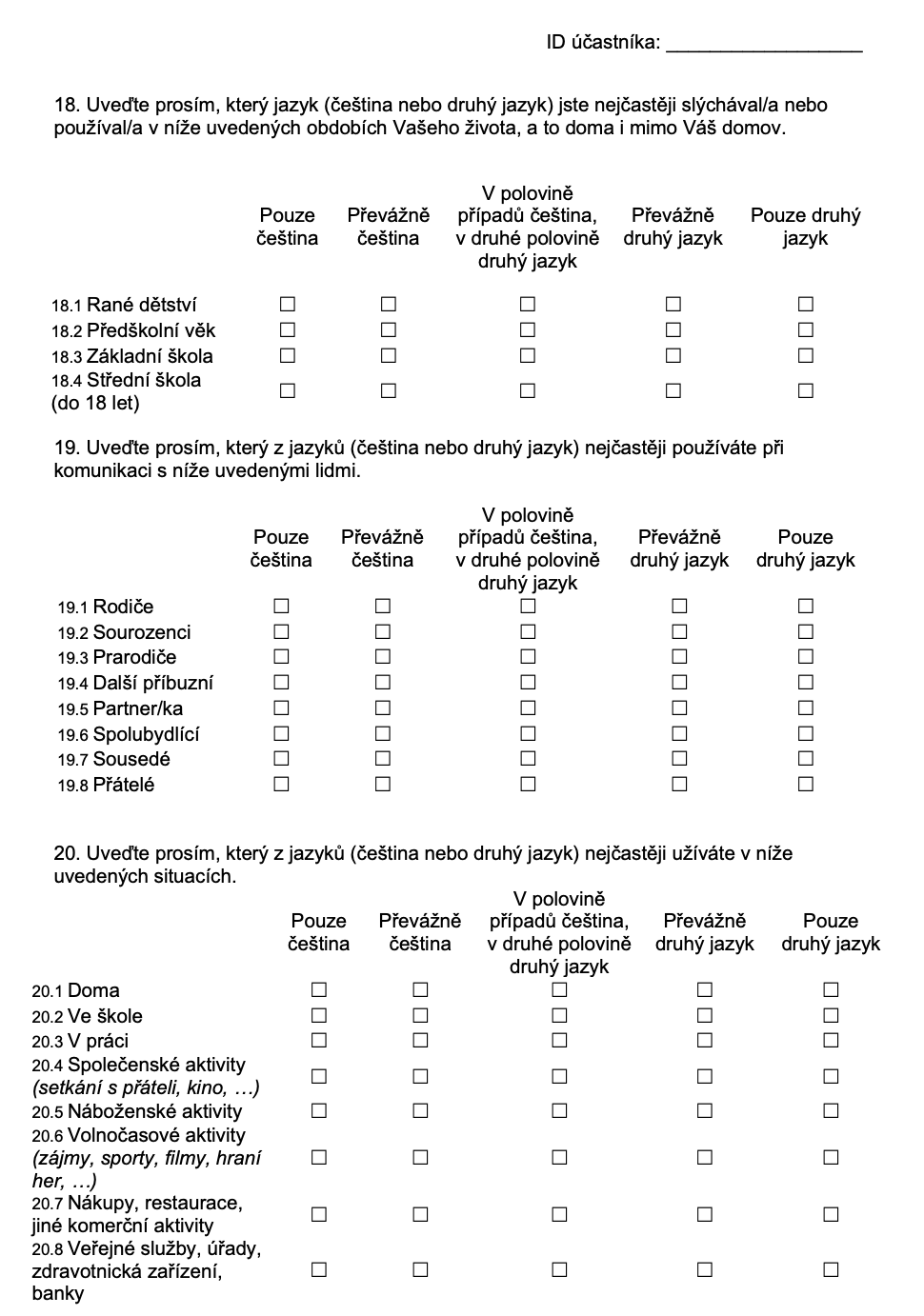


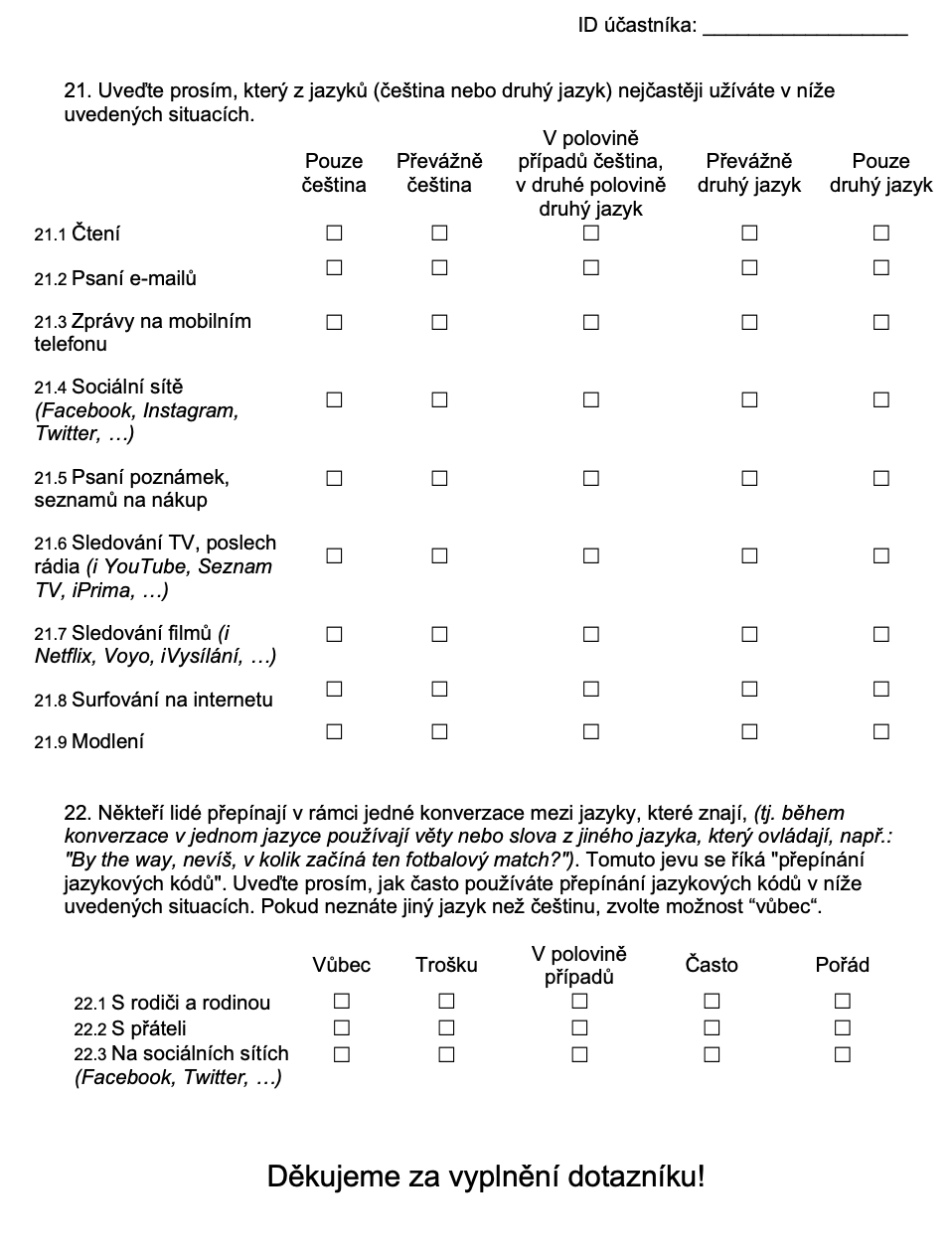

Supplement: Supplementary file 1 — Supplementary Information. [file 41598_2023_30326_MOESM1_ESM.docx]
